# BioTransformer 3.0—a web server for accurately predicting metabolic transformation products

**DOI:** 10.1093/nar/gkac313

**Published:** 2022-05-10

**Authors:** David S Wishart, Siyang Tian, Dana Allen, Eponine Oler, Harrison Peters, Vicki W Lui, Vasuk Gautam, Yannick Djoumbou-Feunang, Russell Greiner, Thomas O Metz

**Affiliations:** Department of Biological Sciences, University of Alberta, Edmonton, AB T6G 2E9, Canada; Department of Computing Science, University of Alberta, Edmonton, AB T6G 2E8, Canada; Department of Laboratory Medicine and Pathology, University of Alberta, Edmonton, AB T6G 2B7, Canada; Faculty of Pharmacy and Pharmaceutical Sciences, University of Alberta, Edmonton, AB T6G 2H7, Canada; Biological Sciences Division, Pacific Northwest National Laboratory, Richland, WA 99352, USA; Department of Biological Sciences, University of Alberta, Edmonton, AB T6G 2E9, Canada; Department of Biological Sciences, University of Alberta, Edmonton, AB T6G 2E9, Canada; Department of Biological Sciences, University of Alberta, Edmonton, AB T6G 2E9, Canada; Department of Biological Sciences, University of Alberta, Edmonton, AB T6G 2E9, Canada; Department of Biological Sciences, University of Alberta, Edmonton, AB T6G 2E9, Canada; Department of Biological Sciences, University of Alberta, Edmonton, AB T6G 2E9, Canada; Corteva Agriscience, Indianapolis, IN 46268, USA; Department of Computing Science, University of Alberta, Edmonton, AB T6G 2E8, Canada; Alberta Machine Intelligence Institute, University of Alberta, Edmonton, AB T6G 2E8, Canada; Biological Sciences Division, Pacific Northwest National Laboratory, Richland, WA 99352, USA

## Abstract

BioTransformer 3.0 (https://biotransformer.ca) is a freely available web server that supports accurate, rapid and comprehensive *in silico* metabolism prediction. It combines machine learning approaches with a rule-based system to predict small-molecule metabolism in human tissues, the human gut as well as the external environment (soil and water microbiota). Simply stated, BioTransformer takes a molecular structure as input (SMILES or SDF) and outputs an interactively sortable table of the predicted metabolites or transformation products (SMILES, PNG images) along with the enzymes that are predicted to be responsible for those reactions and richly annotated downloadable files (CSV and JSON). The entire process typically takes less than a minute. Previous versions of BioTransformer focused exclusively on predicting the metabolism of xenobiotics (such as plant natural products, drugs, cosmetics and other synthetic compounds) using a limited number of pre-defined steps and somewhat limited rule-based methods. BioTransformer 3.0 uses much more sophisticated methods and incorporates new databases, new constraints and new prediction modules to not only more accurately predict the metabolic transformation products of exogenous xenobiotics but also the transformation products of endogenous metabolites, such as amino acids, peptides, carbohydrates, organic acids, and lipids. BioTransformer 3.0 can also support customized sequential combinations of these transformations along with multiple iterations to simulate multi-step human biotransformation events. Performance tests indicate that BioTransformer 3.0 is 40–50% more accurate, far less prone to combinatorial ‘explosions’ and much more comprehensive in terms of metabolite coverage/capabilities than previous versions of BioTransformer.

## Graphical Abstract

**Graphical Abstract:**
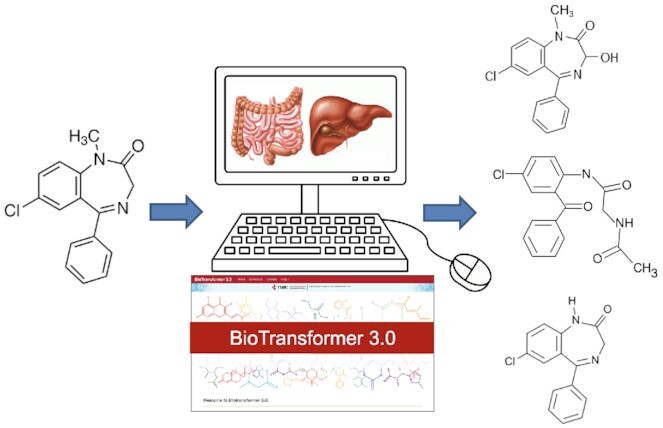
Synopsis of BioTransfomer 3.0 functions.

## INTRODUCTION

Metabolism is typically divided into two broad categories: (i) primary metabolism and (ii) secondary metabolism. Primary metabolism refers to the metabolic processes associated with the production (anabolism) and breakdown (catabolism) of essential metabolites such as nucleic acids, lipids, amino acids and steroids. Primary metabolism is critical to the production of energy and the sustenance of essential cellular processes (growth, cell division, repair, etc.). Secondary metabolism refers to the metabolic processes associated with the production and breakdown of non-essential, xenobiotic, foreign or exogenous chemicals. For humans, secondary metabolism is mostly limited to those processes associated with the catabolism or breakdown or modification (i.e. biotransformation) of chemicals rather than their biosynthesis. This xenobiotic biotransformation process usually involves the activation, detoxification and eventually the elimination of xenobiotics. Xenobiotics are compounds such as drugs, cosmetics and personal care products, clothing dyes, pesticides and herbicides, plant-derived food compounds, food additives, surfactants, solvents, and other synthetic or biologically foreign substances that can be converted into energy or primary metabolites needed for essential cellular processes.

Over the past 20 years, two fields of ‘omics’ science have emerged that are focused on comprehensively characterizing the products of both primary and secondary metabolism: metabolomics and exposomics. Metabolomics uses high-throughput techniques such as mass spectrometry (MS) and nuclear magnetic resonance (NMR) spectroscopy to comprehensively characterize the metabolome (i.e. endogenous molecules) while exposomics uses many of the same methods to characterize the exposome (i.e. exogenous or environmental molecules) (1,2). While metabolomics and exposomics emerged somewhat separately, they have progressively converged over the past 5–10 years. This is because the same analytical methods are often employed, and the same molecules often end up being identified. Indeed, as we learn more and more about the human metabolome (3) and the human exposome (4,5), the only significant differences in their chemical compositions often seems to be in the measured concentrations.

From the experimental perspective both metabolomics and exposomics continue to face similar challenges. Indeed, both fields struggle with the identification of chemical ‘unknowns’. These are the compounds that defy facile identification or which are not found in any known chemical or spectral (MS or NMR) database. These unknowns are often called chemical dark matter (6). Estimates vary but there is general agreement that between 90 and 95% of the signals detected by MS-based metabolomics or exposomics studies correspond to unknown chemicals or chemical dark matter (6,7).

The characterization or identification of unknown compounds from biological or environmental samples is quite difficult. Often it can take months or even years to positively identify a metabolite using standard analytical chemistry techniques. Consequently, there has been a growing interest in using *in silico* strategies to help with this process. The general concept behind these *in silico* strategies is to have a computer predict the metabolic reactions and reaction products from a given query molecule using various expert-derived rules or pattern recognition techniques (8–10). Because of its potential to generate completely novel, but biologically feasible chemical structures, *in silico* metabolism prediction has seen growing interest by researchers in metabolomics and exposomics as it provides testable hypotheses to help identify unknown or previously uncharacterized molecules. *In silico* metabolism is also attracting interest among researchers performing drug metabolism studies, drug safety assessments, food chemistry/food analysis, microbial and plant biochemistry, environmental contaminant analysis and many other areas of life science (7–12).

Most *in silico* metabolism prediction tools are quite specific to certain classes of reactions or certain metabolic or biotransformation processes. Some are limited to predicting the products of secondary metabolism while others are focused on predicting the transformation products of primary metabolism. For instance, SMARTCyp (13,14), isoCYP (15) and ADMET Predictor (https://www.simulations-plus.com/software/admetpredictor/metabolism/) are examples of *in silico* metabolism predictors that predict only human cytochrome P450 (so-called phase I) metabolism. Other *in silico* metabolism predictors, such as Meteor Nexus (10,16) and SyGMa (17), cover both phase I and phase II (such as sulfation and glucuronidation) biotransformations. Some *in silico* metabolism programs are targeted towards predicting the products of environmental microbial degradation, such as enviPath, EAWAG-BBD/PPS or the UM-BBD and UM-PPS systems (11,18). Still others focus on predicting primary metabolism. For instance, the Biochemical Network Integrated Computational Explorer (BNICE) algorithm uses reaction rules based on the Enzyme Commission (EC) classification system to predict promiscuous enzyme metabolic products from query molecules (19). However, the fact that there are so many specialized, single-purpose, *in silico* metabolism prediction systems means that users must learn how to download, install and run different programs and then extract and merge the results to generate useful predictions for metabolomics or exposomics.

This software ‘fragmentation’, combined with the fact that many existing software tools perform inconsistently and others are very expensive, led us to develop our own generalized, free, open-source, open-access *in silico* metabolism predictor called BioTransformer in 2019 (9). The goal of BioTransformer 1.0 was to provide the metabolomics and exposomics community with a fast, accurate, online tool to predict the structure and enzymatic provenance of metabolic byproducts arising from human secondary metabolism. The initial version of the BioTransformer web server was very well-received and feedback from users led to BioTransformer 2.0, which was released in 2020. BioTransformer 2.0 provided a slightly improved web interface and corrected a number of minor backend errors. Here, we describe BioTransformer 3.0, which represents a substantial upgrade to previous versions of BioTransformer. These upgrades include: (i) improved support for both primary and secondary metabolism prediction; (ii) a completely re-written and far more user-friendly interface; (iii) new functionalities and prediction modules and (iv) improved accuracy and performance. These improvements are discussed in more detail below.

## GENERAL DESIGN AND OPERATION

BioTransformer 3.0 is a general-purpose *in silico* metabolism prediction tool. It is mainly intended to predict human (or mammalian) biotransformations arising from primary or secondary metabolism. BioTransformer 3.0 also supports the prediction of environmental (i.e. soil/water) biotransformations of man-made chemicals. BioTransformer 3.0 can be used to not only predict the structures of metabolites or metabolic byproducts, but it can also assist with the identification of molecules through SMILES (20) string filtering, molecular formula filtering and/or windowed mass filtering based on the calculated structures, chemical formulas or masses of the predicted products. BioTransformer 3.0’s output also provides information on the enzymatic or biological provenance and reaction processes responsible for the predicted products.

**Figure 1.**
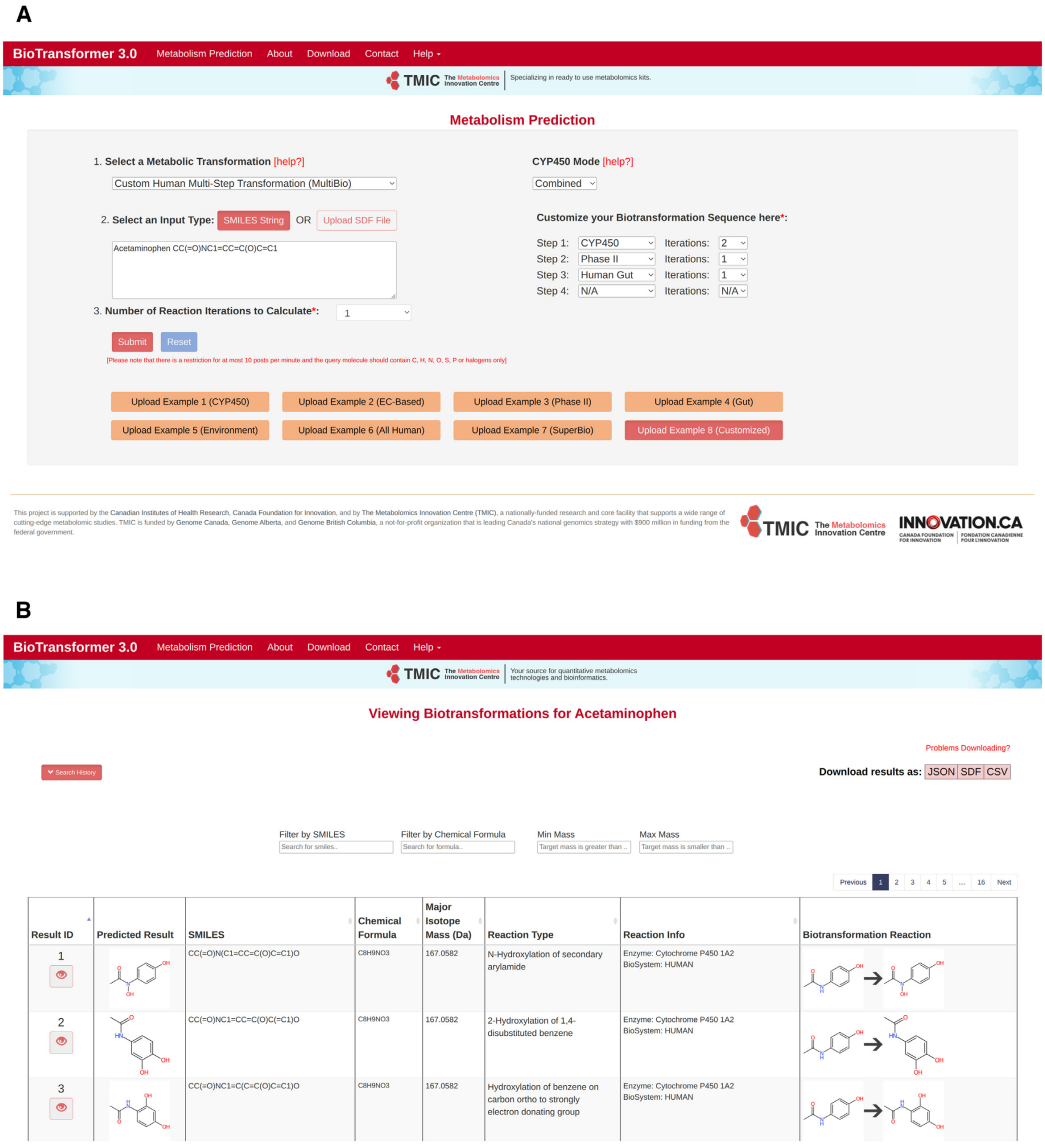
Screenshots of the (**A**) **Metabolism Prediction** page and (**B**) the **Biotransformation Viewer** page with examples of typical user input and output. The **Metabolism Prediction** page serves as BioTransformer's data input page while the **Biotransformation Viewer** page serves as the web server's output page. For this example (panel A), a customized metabolic prediction (*MultiBio* – Example #8, highlighted) has been chosen. As seen here, users must select or fill in specific information about the type of Metabolic Transformation, the molecule (acetaminophen), the CYP450 Mode (Rule-based only, CyProduct only or Combined), and the Biotransformation sequence. The output from the calculation is shown (in panel B) to illustrate the type of information generated and the format in which it is presented. Many of the columns in the table are sortable and the table itself is filterable. Links to the downloadable files (JSON, CSV, SDF) are located in the top right corner. As this particular query generated many acetaminophen metabolites, users can jump from page to page to view these predictions using the navigation tool at the top (or bottom) of the table.

BioTransformer 3.0 is very simple to use. Users submit a single molecular structure as input (using SMILES or an SDF file) and the web server outputs an interactively viewable/sortable table of the predicted metabolites or transformation products along with the reaction equation(s), the reaction types and the enzymes or biological processes that are predicted to be responsible for those reactions. BioTransformer 3.0 will accept most types of small molecule structures as input but the molecule must be organic, it should have a molecular weight <1500 Da and it must not be a mixture. In addition to producing an easily navigated output data table, a variety of downloadable files (in JSON, CSV and SDF formats) containing additional or more detailed metabolite prediction information are also available. BioTransformer's calculations typically take less than a minute but for more complex biotransformation predictions with more iterations and/or with much larger molecules, these calculations can take up to 30 minutes. Screenshots of BioTransformer's **Metabolism Prediction** page and a typical output page for BioTransformer's **Biotransformation Viewer** page (for acetaminophen as the query molecule) are provided in Figure 1.

The user interface for BioTransformer 3.0 has been completely redesigned. Therefore, a brief summary of its layout and operation is certainly warranted. To begin a BioTransformer session, users must first go to the BioTransformer 3.0 website (https://biotransformer.ca). From here, users can press the red ‘Metabolism Prediction’ banner on the home page to start a prediction session. This takes the user to the **Metabolism Prediction** page. At the top of the page, there are five menu items: **Metabolism Prediction**, **About**, **Download**, **Contact** and **Help**. Selecting **Metabolism Prediction** allows users to easily perform additional predictions without having to go to the home page. Clicking **About** produces a web page that provides a brief summary of BioTransformer's functions, its overall design, implementation, evaluation/performance and examples of the types of biotransformation predictions it can perform. Selecting **Download** allows users to go to a page with instructions on how to download and install a command-line version of BioTransformer 3.0 through the (Bitbucket) hyperlink listed on the page. Details about the computer requirements and installation/operation process are also provided. The **Help** link provides a submenu (Overview, Metabolism Prediction, Metabolite Identification, Programmatic Access and Browser Compliance) with written details and screenshots to help explain each of the different topics or features.

To perform metabolism prediction with BioTransformer, users must first select a specific **Metabolic Transformation** from the pull-down menu at the top of the **Metabolism Prediction** page. BioTransformer supports eight types of metabolic transformation predictions including phase I reactions (cytochrome P450), promiscuous enzymatic (EC) reactions, phase II reactions, human gut microbial reactions, environmental microbial transformations and different combinations of the above, known as *AllHuman*, *SuperBio* and *MultiBio*. More specifically, the ‘*Phase I (CYP450) Transformation*’ option predicts cytochrome P450 metabolism. In humans, CYP450 metabolism is commonly done by nine major CYP450 enzymes. These enzymes play a very important role in drug or xenobiotic metabolism by performing various oxidation or hydroxylation reactions to increase the water solubility of the substrate. The ‘*EC-Based Transformation*’ option predicts regular and promiscuous enzymatic metabolism which mostly corresponds to primary metabolism. Predicted metabolites are generated using reaction rules associated with different enzymes specified by EC numbers. The ‘*Phase II Transformation*’ option predicts major conjugative reactions, including glucuronidation, sulfation, glycination, N-acetylation, and glutathione transfer, among others. These reactions also play important roles in xenobiotic or secondary metabolism, elimination and detoxification. The option ‘*Human Gut Microbial Transformation*’ predicts metabolism arising from gut microbial enzymes using known reaction types and bacterial enzymes found in human gut microbes. Gut microbes perform a wide range of unusual catabolic reactions on both primary and secondary metabolites. The ‘*Environmental Microbial Transformation*’ option predicts metabolites arising from environmental microbial degradation of small organic molecules and is intended for predicting the breakdown products of xenobiotics and secondary metabolites left in soil or water or exposed to light.

Three ‘combination’ transformation options are also offered: *AllHuman*, *SuperBio* and *MultiBio*. The ‘*AllHuman*’ option predicts biotransformations occurring both in human tissues (Phase I, Phase II and EC-based) as well as the gut microbiota. It is intended to mimic general human metabolism without a specific sequence of transformation events. The ‘*SuperBio*’ option also predicts biotransformations occurring both in human tissues and the gut microbiota. Specifically, it runs the *AllHuman* metabolism prediction through four iterations or until no novel metabolites are produced. If users wish to run *SuperBio* metabolism through more than four iterations, they can download and run the executable version of BioTransformer 3.0, which is available on the **Download** page. Finally, the ‘*MultiBio*’ option allows users to create a customized biotransformation sequence (a maximum of four types) by choosing any combination or sequence of CYP450, Phase II, EC-Based, Gut Microbial and Environmental reactions for up to three iterations each. Clicking on the red ‘Help?’ hyperlink provides a detailed explanation of these options. Depending on which **Metabolic Transformation** option is chosen, additional text boxes or pull-downs will appear that must be filled in by the user.

After a **Metabolic Transformation** has been chosen, users must indicate how (i.e. which format) their input molecule will be entered using **Select an Input Type**. Users may choose to type or paste a single SMILES string into the text box or they may select to upload a single SDF file containing only one structure. Choosing the SDF option (**Upload SDF File**) creates a new web form where users must choose a local SDF file on their computer using a standard file browse. As noted earlier, BioTransformer will accept most types of small molecule structures as input, but the molecule must be organic, it must contain carbon, hydrogen, nitrogen, oxygen, phosphorus, sulfur (CHNOPS) and halogen atoms only, and it should have a molecular weight <1500 Da. BioTransformer is capable of handling or metabolizing a number of small biopolymers including small peptides, small polynucleotides, polysaccharides, polymerized polyphenols and phenolic glycosides. Once the SMILES string or SDF file has been uploaded, users must choose the **Number of Reaction Iterations to Calculate**. This is a pull-down option that allows users to choose from one (the default) to three (the maximum) reaction iterations. Reaction iterations refer to the number of times the chosen *in silico* reaction will be performed. If one chooses two iterations, the reaction products of the first reaction will be fed back into the selected reaction scheme for BioTransformer to predict further transformations.

After filling out the **Metabolism Prediction** submission form, users can press **Submit** and the calculations will be performed. If an error has been made during the data entry process, users can always press **Reset** to clear the form. The **Metabolism Prediction** page also lists eight different biotransformation examples (the orange buttons at the bottom of the page—see Figure 1A) that users can select to test the server or to learn more about the operation or input expectations for different **Metabolic Transformation** options. These examples ‘autofill’ all the required data fields on the page and cover different molecules such as acetaminophen and D-glucose. When the **Submit** button is pressed, users are taken to a different page which indicates the progress of the calculation. Most calculations submitted by most users will take less than a minute. For long or complex calculations (*SuperBio*, *AllHuman* or reactions with multiple iterations) users may bookmark the page and return after a few minutes. If users wish to perform transformation predictions on many molecules or if they routinely perform complex transformation predictions, we strongly recommend that they download and install a local, command-line version of BioTransformer and run it on their own computer.

As indicated, once users press the **Submit** button, they are taken to a new page that indicates the progress of the calculation. This is shown along with summary information about the input structure and selected calculation parameters. After the calculations are complete an output page or **Biotransformation Viewer** is generated (see Figure 1B). This page contains a scrollable, interactive, HTML-formatted table listing each predicted product (a PNG file of the structure), the SMILES string, the chemical formula, the molecular weight, the reaction type (responsible for the reaction), reaction information (naming the specific enzyme or biological process), along with an image showing the biotransformation reaction (reactant and product). Users may sort most of the columns by clicking on the up/down arrows at the top of each column. At the top of the **Biotransformation Viewer**, we have provided ‘Search History’ button which users can click to see their original search query. The **Biotransformation Viewer** page has four fillable text boxes. These allow users to filter or select the results according to the structure (using the SMILES filter), the chemical formula or the molecular weight ranges. Both the SMILES and chemical formula filters require exact matches. The chemical formula must be in the standard CHNOPS order. These filtering functions are particularly useful if the biotransformation prediction generates dozens to hundreds of possible metabolites where the resulting products are similar in structure or isomeric. They are also useful for metabolite identification (in metabolomics and exposomics studies) as users may use accurate mass matching or derived chemical formula matching (from high resolution mass spectrometry) to identify possible hits.

In addition to the browsable product table presented through the **Biotransformation Viewer**, users can also download BioTransformer's results in several different formats (SDF, JSON and CSV). All the predicted structures are available in SDF formatted files, which are obviously much larger and more detailed than SMILES or InChI files. Additionally, the JSON and CSV files contain the predicted structures in SMILES, InChI and InChIkey formats. The generated JSON and CSV files also contain additional information that is not displayed in the more simplified **Biotransformation Viewer**. In particular, these downloadable files include the names and synonyms of the generated compounds (if available), their PubChem IDs (if available), the enzymatic biosystem (human, microbial, environmental) associated with the transformation(s), as well as the product and precursor ALogP values. Users can obviously use the JSON or CSV files for further offline processing and manipulation.

While the frontend of BioTransformer 3.0 is designed to be intuitive and very simple to use, it actually hides a very complex and sophisticated backend. Many of the details of BioTransformer's backend and how the components were implemented, tested and integrated were provided in the initial description of BioTransformer 1.0 (9). However, several changes and additions have been made and these are briefly outlined here. Currently, the backend of BioTransformer 3.0 consists of a dedicated Metabolism Prediction Tool (MPT) consisting of several predictive software tools including CypReact (21), CyProduct (22) and HMDBMetabolizer, as well as a reaction database called BTKB (BioTransformer KnowledgeBase). CypReact is a program that uses machine learning to predict whether a molecule will be a substrate for one or more of the nine most common human cytochrome P450 enzymes. CyProduct, on the other hand, is a program that uses the output from CypReact in combination with machine learning algorithms to predict the bonds of metabolism and reaction products arising from a compound that is a substrate for the nine common human cytochrome P450 enzymes. HMDBMetabolizer is a separate program for predicting primary metabolism of endogenous molecules. It compares BioTransformer query compounds to endogenous (i.e. primary) metabolite structures in the HMDB. If a match is found, it uses the reactions, substrates and reaction products in HMDB to generate the product structures and to annotate the reactions accordingly. HMDBMetabolizer is conceptually similar to BTKB but is intended to handle primary metabolism or primary metabolite prediction instead of secondary metabolite prediction or xenobiotic metabolism.

BioTransformer's reaction knowledge database (BTKB) consists of >900 reaction rules primarily intended for xenobiotic, secondary or exogenous compound metabolism. These rules were derived from extensive literature searches and implemented as carefully validated, hand-crafted SMARTS (23) and SMIRKS (24) strings. Each biotransformation in the BTKB includes a starting reactant (structure and identifiers), a reaction product (structure and identifiers), the name or type of the enzyme catalyzing the biotransformation, the type of reaction, and one or more citations. In BioTransformer, a reactant is defined as a small molecule that binds to a specific enzyme and undergoes a reaction catalyzed by that enzyme while a reaction product is a small molecule that arises from a specific enzymatic or a general biologic reaction. In contrast to most reaction rules in the BTKB, which are strictly enzyme-based, the environmental microbial metabolism resources in the BTKB use handwritten SMARTS/SMIRKS rules for less well-defined or multi-enzyme ‘biological processes’ that were manually extracted and tested from the enviPath system (25).

## USER INTERFACE IMPROVEMENTS

Some of the most significant changes and improvements to BioTransformer 3.0 were made to its user interface. Earlier versions of BioTransformer had query and output viewing pages that many users noted were not particularly intuitive. The output page (i.e. the **Biotransformation Viewer**) was hard to navigate and provided very limited information about the predicted metabolites and their reactions or provenance. Likewise, its layout differed radically from the layout and design of the home page and query (i.e. the **Metabolism Prediction**) page. This gave the appearance that different parts of the web server were built completely independently of each other. Furthermore, the information about how to operate the server and explanations about its design, performance, input requirements and capabilities were minimal or largely absent. In addition, the number of examples with which users could test the server was quite minimal and did not span all possible queries or query types.

Given the many issues related to BioTransformer 1.0’s design and usability, a complete redesign of its graphical user interface (GUI) was undertaken. For BioTransformer 3.0 the home page, the **Metabolism Prediction** page and the **Biotransformation Viewer** page were significantly modified to match the style, layout and design of our other popular web servers and web-based databases such as HMDB (3), CFM-ID (26), and NP-MRD (27). These layouts are time-tested, easy to understand and well-liked by many thousands of users. Additionally, much more extensive **About** and **Help** sections were written which included annotated screenshots and far more detailed explanations or instructions. An intermediate progress screen was also added so that users could be more certain that the calculations were proceeding as expected. As noted before, BioTransformer's query interface or **Metabolism Prediction** page was also simplified and the option to have multiple metabolites processed at once (which led to long queues and processing times running for hours) was removed. Furthermore, the BioTransformer Metabolite Identification module (BTMI) was eliminated from the query interface and, instead, new functions were added to the **Biotransformation Viewer** page to allow users to perform metabolite identification through the SMILES/structure, chemical formula, and molecular weight filters. This modification was done to improve usability and simplify the **Metabolism Prediction** page.

The most important interface changes for BioTransformer 3.0 were for the **Biotransformation Viewer**. Rather than having multiple tabbed views or displaying hard-to-understand InChI strings (instead of more understandable SMILES or structure images) or having numerous options to reformat the table of predicted metabolites, the layout was redesigned and simplified so that all the most relevant information (the predicted product structure, the SMILES string, the chemical formula, the molecular weight, the reaction type, the reaction information along with an image showing the biotransformation reaction) could be viewed in a single table. Users can click on the ‘eye’ icon in the first column of the result table to generate a detailed result card for the selected biotransformation. Each card contains details such as the synonyms, InChI string, InChIKey, molecular formula, major isotope mass, AlogP etc. These details are also available to the user as JSON and CSV files in the download section. Furthermore, to facilitate metabolite identification and comparison, new table sorting utilities and filtering functions for structure, chemical formula, and mass range have been added. Similarly, the quality of the structure renderings and chemical reaction renderings was significantly improved. Users can also click on the structure image, which is then rendered as an expanded modal view. This allows users to more closely inspect the structure and to download the image as an SVG file. Additionally, the amount of information available in the JSON and CSV files about each of the predicted metabolites and their provenance was substantially increased. Overall, we believe these user interface improvements should significantly improve the usability, stability and user friendliness of this web server.

## BACKEND IMPROVEMENTS

The focus of most of the backend improvements to BioTransformer 3.0 was on improving performance, functionality, accessibility, and speed. From the performance perspective there are at least four major improvements worth noting. One of the more important enhancements involved the addition of CyProduct to the BioTransformer system. Cytochrome P450 metabolism and metabolites are notoriously difficult to predict. Older versions of BioTransformer used rule-based predictions built as SMIRKS strings into the BTKB. While very fast to calculate, these were not particularly accurate. In particular, we compared the CYP450 metabolism module in BioTransformer 3.0 with the previous version of BioTransformer using a dataset of 68 well studied reactants (as previously described in CyProduct (22)). Each reactant and its CYP450 enzyme-specific metabolites have been previously reported in a variety of drug metabolism publications (the complete collection is downloadable from BioTransformer's **Download** page). We then manually checked the BioTransformer-predicted metabolites (version 2.0 versus version 3.0) against the known, published metabolites for each substrate-enzyme pair. As shown in Table 1, the accuracy of the CYP450 metabolism module in BioTransformer 3.0 was substantially improved over BioTransformer 2.0 by incorporating CyProduct into the metabolite prediction pipeline. However, because the old rule-based method is slightly faster and tends to over-predict metabolites (some of which can appear in nature), and CyProduct tends to be more cautious (but more accurate), users now have the option of choosing CyProduct alone, the rule-based method alone or the merged combination of both.

**Table 1.** Comparison between CyProduct, ADMET Predictor and BioTransformer's older rule-based Cyp450 metabolism predictor. The performance of each program was evaluated over 68 well-studied Cyp450 reactants for the 9 most common human cytochrome P450 enzymes (Cyp 1A1, Cyp 2A6, etc.) using the Jaccard score to evaluate correct and incorrect predictions. More details about this evaluation set are available in reference (22). If no distinction between cytochrome P450 enzymes is made the collective or average performance of each method improves by 20–25%

	Cyp 1A2	Cyp 2A6	Cyp 2B6	Cyp 2C8	Cyp 2C9	Cyp 2C19	Cyp 2D6	Cyp 2E1	Cyp 3A4
BioTransformer 3.0 (CyProduct)	0.471	0.391	0.333	0.263	0.429	0.385	0.511	0.611	0.366
ADMET Predictor	0.381	0.318	0.276	0.042	0.206	0.250	0.338	0.190	0.299
BioTransformer 2.0 (Rule Based)	0.113	0.263	0.200	0.152	0.128	0.125	0.121	0.125	0.112

Another backend performance improvement for BioTransformer 3.0 involved the introduction of new software and new transformation rules (in the BTKB) to handle small biological polymers such as small peptides, small polynucleotides, polysaccharides, polymerized polyphenols and phenolic glycosides. In particular, BioTransformer 3.0 is now able to recognize these types of biopolymers and to cleave or depolymerize them appropriately. Prior to the addition of this module, BioTransformer would often metabolize these biopolymers inappropriately and generate unrealistic or biologically infeasible metabolites. In many cases, older versions of BioTransformer would generate a combinatorial explosion of multiple Phase I and Phase II metabolites for simple biopolymers, leading to multi-hour processing times and long, useless lists of thousands of incorrect or unlikely metabolites.

Yet another important performance improvement involved the introduction of statistical methods to prevent other kinds of combinatorial metabolic explosions or interminable metabolic reactions from taking place. Specifically, we introduced probabilistic models that are based on HMDB data about human urinary metabolites, fecal metabolites and blood metabolites. This allows BioTransformer to predict if a compound is physico-chemically more similar to known liver/kidney metabolites (which are more abundant in urine) or gut microbial metabolites (which are more abundant in feces). If a predicted metabolite generated by BioTransformer 3.0 is not sufficiently similar, as measured by various physico-chemical criteria, to known liver/kidney or gut microbial metabolites, the biotransformation reaction that produced that metabolite is aborted and the precursor compound will serve as the biotransformation end product. This algorithm appears to substantially reduce excessive metabolism of many compounds and generates results that more closely match known metabolite datasets from well-studied molecules such as nicotine, acetaminophen and epicatechin.

Other performance improvements were achieved by correcting various bugs or programming errors in BioTransformer's original reaction databases or algorithms. These included fixing a bug that removed small metabolite byproducts (such as acetate or CO_2_) as well as removing duplicate biotransformations in the BTKB. Through extensive testing, several other bugs were identified and corrected that would occasionally send BioTransformer into an infinite loop. A total of 17 incorrect SMIRKS strings were corrected and another 15 SMIRKS strings were added to accommodate new reactions. Another bug that mishandled SMILES queries with specific stereo specifications was also corrected. Likewise rewrites of the *AllHuman* and *SuperBio* modules were done to improve both the efficiency and performance of these modules.

In terms of functional improvements to BioTransformer 3.0 there are at least two enhancements of note. One is the development of HMDBMetabolizer. This module was developed to support the proper handling of primary metabolites and the proper prediction of their metabolism or metabolic byproducts. Prior to the introduction of this module, BioTransformer would treat these compounds as xenobiotics and would often generate biologically infeasible or non-sensical metabolites. Algorithmically, HMDBMetabolizer compares any BioTransformer query compound to a list of all known endogenous (i.e. primary) metabolite structures in the HMDB. If an exact match is found, it uses the reactions, substrates and reaction products in HMDB to generate the product structures and annotate the reactions accordingly.

The other major functional improvement introduced to BioTransformer 3.0 is the customizable biotransformation module called *MultiBio* or the Custom Human Multi-Step Transformation module. This option allows users to create a customized biotransformation sequence (a maximum of 4) by choosing any combination or sequence of CYP450, Phase II, EC-Based, Gut Microbial and Environmental reactions with up to three iterations for each step and up to four iterations overall. For example, if a user selects CYP450 with 2 iterations for step 1, Gut Microbial with 1 iteration for step 2, Phase II with 1 iteration for step 3 and N/A for step 4, BioTransformer will generate all the metabolites for the given query molecule arising from one round of CYP450 transformation along with the CYP450 metabolic products of all those CYP450 metabolites. Then all those doubly-processed CYP450 metabolites will be processed via human gut microbial metabolism, whereupon all of those metabolites (which may now number in the dozens) will go through a single round of Phase II metabolism. Clearly it is possible to generate dozens to hundreds of metabolites from a single precursor through this custom biotransformation module. Overall, we believe these functional improvements to BioTransformer 3.0 should greatly improve its utility and coverage.

In terms of accessibility improvements, there have been two notable enhancements with this latest release of BioTransformer. One has been through the addition of an improved API (application programming interface) that allows BioTransformer users to programmatically access the BioTransformer 3.0 web server to perform automated queries. Currently the number of post requests is limited to 2 per minute. Details on how to use the API along with screenshots and example code are provided under the **Help** section and the submenu entitled ‘Programmatic Access’. The second enhancement has been directed towards improvements of the command-line version of BioTransformer. The JAR version of this program, which contains essentially all of the predictive tools and options in the web server, can be downloaded from Bitbucket (the URL link and installation information is provided under **Download**). Significant improvements have been made to simplify the installation, robustness, performance, customizability and compatibility of the command-line version. Likewise, more information has been provided on the website to facilitate installation and operation of the program. The improved accessibility of the command-line version of BioTransformer 3.0 should allow users to perform large-scale biotransformation studies on their local computers and to more easily process data from metabolomic and exposomic experiments.

Finally, BioTransformer has also had some of its modules optimized for speed. For instance, in order to accelerate the prediction process, a new feature has been implemented that allows BioTransformer 3.0 to retrieve previously predicted results submitted from other users. Given the frequency with which the same molecules and the same transformation schemes are submitted to BioTransformer, we expect this feature will be heavily used. The rewrites of the *SuperBio* and *AllHuman* modules along with modifications to the logic workflow of CyProduct have led to speed-ups of up to 20× for some of these modules. The performance improvement involving CyProduct was particularly important as it is now comparable in speed to the (less accurate) rule-based CYP450 metabolism predictor. Had CyProduct's speed not been significantly optimized, it is unlikely users would have opted to use this module.

## IMPLEMENTATION

The backend for BioTransformer 3.0 was implemented in the Java programming language. The command-line version (as a JAR file available via Bitbucket and the **Download** link) is compatible with Linux, Mac OS X, and Windows. BioTransformer also uses two other open-source tools, namely the Chemistry Development Kit (CDK) (28) and the AMBIT library (23,24). CDK is used for several operations, including the calculation of physico-chemical properties, the execution of superstructure search operations, and the handling of chemical structures. The AMBIT library is used for the application of biotransformation rules and structure generation. The frontend of BioTransformer has been implemented as a RESTful web service using the JRuby on Rails framework. Ruby on Rails is a development system that employs a concept called the Model-View-Controller (MVC). In the MVC framework, models respond and interact with the data, views create the interface to show and interact with the data, and controllers connect the user to the views. This framework has allowed the BioTransformer programming team to rapidly develop, prototype and test all of BioTransformer's web modules and page views. The search utilities (SMILES, mass or chemical formula searches) and table sorting utilities are borrowed from a large collection of Ruby gems previously developed for the HMDB (3). The data used to train, test and evaluate many components of the BioTransformer 3.0 web server are available in the published papers and supplementary material describing BioTransformer 1.0 (9), CypReact (21) and CyProduct (22).

## CONCLUSION AND FUTURE PLANS

Overall, we believe the enhancements, additions and improvements to both the backend and the frontend of BioTransformer 3.0 should make it easier to use, faster, more user-friendly, much more accessible, far more stable, more broadly usable and significantly more accurate. Likewise, the availability of a much more robust and stable, downloadable version of BioTransformer 3.0 should help address the needs of those users demanding much higher throughput or larger scale biotransformation analyses. While the improvements we have enumerated here are significant, there are still some shortcomings and limitations to BioTransformer 3.0 that we would like to address in the future. Some of these suggestions have come from regular BioTransformer users while others have emerged from our own investigations. These include the following:

1. The incorporation of new rules or the development of new machine learning algorithms to better predict sulfation reactions (a common Phase II biotransformation).
2. The integration of more diverse gut microbial metabolism reactions and enzymes, especially those that have arisen from many recent gut metagenomic and gut microbial metabolomics studies.
3. Improvements to the SMIRKS rules (number and type) in the BTKB to better handle known pesticide and herbicide breakdown reactions—both in mammals and in the environment.
4. The addition of new reaction rules to handle water decontamination byproducts arising from chlorination, ozonation and ultraviolet radiation.
5. Developing better methods (statistical or machine learning) for recognizing when biotransformation reactions should be terminated.
6. Improving the overall accuracy of Phase I and Phase II metabolism prediction.
7. Developing probability scores to indicate the likelihood of a reaction occurring or a product existing.

No doubt there are many other ideas that could/should be pursued, and we certainly invite the community to contribute to the discussion. Indeed, the field of *in silico* metabolism is still very much in its infancy and so there is still plenty to do to improve it. In this regard, it is also important to remember that the primary role of tools such as BioTransformer is to be an idea generator or a hypothesis generator. The molecules generated by BioTransformer 3.0 are biologically feasible, but they are not biological certainties. Indeed, the accuracy of *in silico* metabolism prediction has not yet reached a point that a given prediction guarantees the existence of a proposed molecule. Likewise, continuing challenges in experimental metabolite extraction and experimental metabolite identification mean that many biotransformed metabolites that really do exist in nature have not been (and may never be) formally identified. This means that the training and testing data needed for improving *in silico* metabolism prediction tools will always be less than perfect. Given that we will never have ‘perfect knowledge’ of all the transformed molecules existing in humans (or in nature), or their provenance, it likely means that we will have to live with the fact that some things in life will always remain a mystery.
